# Selective (dis)honesty: Choosing overly positive feedback only when the truth hurts

**DOI:** 10.1111/bjso.70020

**Published:** 2025-11-23

**Authors:** Katarzyna Cantarero, Michał Białek

**Affiliations:** ^1^ SWPS University Wroclaw Poland; ^2^ School of Psychological Science University of Bristol Bristol UK; ^3^ Uniersity of Wroclaw Wroclaw Poland; ^4^ Uniersity College of Professional Education Wroclaw Poland

**Keywords:** character judgement, feedback, moral judgement, prosocial dishonesty

## Abstract

In two studies (*N* = 886), we examined how individuals judge and select feedback providers for those who either handle criticism well or poorly after performing a low‐quality task. Prosocial liars who provided overly positive feedback, were judged as more moral than honest feedback providers. However, despite this, honest feedback providers were preferred for both oneself and others. Interestingly, when choosing a feedback provider for a vulnerable recipient versus a generic other, participants preferred a prosocial liar in the former case. Similarly, a ‘sensitive’ feedback provider, defined as someone who tells the truth to individuals who handle criticism well but offers overly positive feedback to those who struggle, was also favoured when the recipient was vulnerable compared with when the recipient was unspecified. Notably, the sensitive provider was not judged as less moral than the honest one, suggesting that inconsistent (dis)honesty is tolerated when it aligns with social needs. These findings indicate that individuals strategically adjust preferences for honesty versus lying based on social cues.

Honesty is widely valued (Schwartz, 1994), and those who are truthful often benefit from positive character judgements (Pontari & Schlenker, 2006). Individuals tend to favour honest feedback providers, perceiving them as reliable and predictable. Predictability increases perceptions of morality, whereas behavioural inconsistency reduces them (Turpin et al., 2021). However, in some contexts, strategic inconsistency may be perceived as more moral than rigid honesty.

Consider two cooks, Amy and Kate, who have each prepared an unsuccessful dish. Kate responds well to criticism and uses it to improve, while Amy struggles with negative feedback and finds it demotivating. In such cases, a more nuanced approach may be appropriate: providing direct, constructive feedback to Kate while softening criticism for Amy. This dilemma raises fundamental questions about feedback: Do people prefer feedback providers who are consistently honest, regardless of context? Or do they favour those who adapt their approach based on the recipient's emotional resilience? Moreover, how do individuals evaluate the morality of feedback providers who shift between honest and prosocially tailored, but dishonest feedback? The present research examines how individuals judge and choose feedback providers who exhibit different patterns of honesty and prosocial dishonesty.

Honesty is often regarded as the best policy, and individuals who consistently tell the truth are typically judged as moral (Jensen et al., 2024). Advocates of unconditional honesty argue that prioritizing truthfulness helps prevent harmful deception, even if certain lies may seem benign. Providing honest feedback allows recipients to recognize weaknesses and improve their performance. At a broader level, organizations also benefit from truthful feedback, as it contributes to enhanced employee performance and overall productivity (e.g., Kluger & DeNisi, 1996; London et al., 1999). In that sense, telling the truth should always be preferred over lying. Indeed, in line with Truth‐Default Theory, most information that we communicate to others is true, and we expect others to be truthful as well (Levine, 2014).

However, individuals often face a conflict between honesty and other competing motives, with benevolence being a prominent one. Delivering harsh truths can sometimes cause emotional harm, which is perceived as aversive and morally troubling (Gray et al., 2012). In such contexts, prosocial lies may be viewed as the lesser of two evils and preferred over potentially hurtful honesty. Consequently, individuals may at times evaluate prosocial lies, when intended to benefit the recipient, as even more moral than truth‐telling (Levine & Schweitzer, 2014). When individuals recognize that someone is struggling emotionally or is highly sensitive to criticism, they may prefer to provide overly positive feedback (Lupoli et al., 2017). While this may seem counterintuitive, it can be a rational choice. For instance, an individual who is already experiencing significant stress may struggle to process and act upon negative feedback effectively. In such cases, receiving harsh but accurate feedback, rather than improving performance, may instead cause further emotional distress. Similarly, when individuals aspire to achieve a goal but struggle with negative feedback, others may choose to shield them from harsh honesty to maintain their motivation (Cantarero et al., 2022). As this example shows, one of the primary barriers to feedback receptivity is an individual's sensitivity to threats to their self‐concept (Fulham et al., 2022). Prosocial dishonesty can be perceived as both a compassionate and effective strategy. Telling a prosocial lie may signal prosocial motivation and empathic concern for another individual. Research on moral judgement suggests that the attribution of motivation is central to moral character evaluation (e.g., Carlson et al., 2022). When the recipient's sensitivity to feedback is made salient, individuals are likely to infer prosocial motives on the part of the feedback provider (i.e., sparing the individual from harm), which in turn should enhance positive moral character judgements. Moreover, empathy has been shown to relate positively to telling lies intended to benefit others or protect them from harm (Cantarero et al., 2018). It is therefore likely that individuals judge prosocial liars favourably because they perceive them as high in trait empathy.

However, the psychology of moral evaluations of prosocial lies is more complex. Although empathy is often seen as a marker of moral and socially desirable behaviour, it can sometimes conflict with fairness because it leads to treating certain individuals better than others (e.g., parochial morality, Decety & Cowell, 2015; identifiable victim effect, Kogut & Ritov, 2005; Small et al., 2007). Given this tension, empathic prosocial liars may, in some cases, be evaluated more harshly.

What happens when a feedback provider is sometimes honest and sometimes dishonest? At first glance, such inconsistency might be perceived as a flaw, making the provider appear unreliable and unpredictable. Consistency is generally valued, as it fosters trust (Nowak et al., 2023). At the extreme, consistent and predictable harm is preferred to smaller, but unpredictable harm (Walker et al., 2021). A person who alternates between honesty and dishonesty might be seen as untrustworthy, neither fully honest nor fully prosocial. However, strategic inconsistency can also signal social awareness. The ability to adapt to context, by considering the emotional needs of different recipients, may be seen as a valuable trait. Atari et al. (2023) argue that fairness, one of the core moral foundations, is better conceptualized as two distinct foundations: equality (treating others similarly and distributing resources evenly) and proportionality (allocating rewards and treatment based on individual circumstances and contributions). From this perspective, a feedback provider who consistently tells the truth or consistently offers prosocial lies behaves in line with the equality moral foundation. In contrast, a provider who tells the truth to resilient individuals while softening criticism for vulnerable ones acts in accordance with the proportionality foundation.

Building on this reasoning, we hypothesized that inconsistent, sensitive feedback providers would be judged as more moral than individuals who rigidly adhere to either honesty or dishonesty. The key to effective feedback may lie in knowing when to apply which strategy. However, given the high value placed on honesty (Schwartz, 1994), we expected the truthful feedback provider to be judged slightly less moral than the sensitive feedback provider, followed by the prosocial liar, who consistently gives overly positive feedback regardless of the recipient's sensitivity. Someone who provides honest feedback to a vulnerable individual while lying to a resilient one misapplies both strategies, rendering their approach socially inappropriate. We anticipated that the insensitive feedback provider, who offers overly positive feedback to individuals who handle criticism well but provides honest feedback to those who struggle with criticism, would be perceived as the least moral.

Moral character judgements are shaped by actions that signal traits such as integrity and empathy (Uhlmann et al., 2015), and these judgements strongly influence interpersonal preferences. When individuals perceive a particular feedback provider as moral, they are more likely to prefer interacting with that person. People tend to view others' traits as relatively stable (correspondence bias; Jones & Harris, 1967; Gilbert & Malone, 1995) and often overestimate the negative impact of honest feedback on recipients (Levine & Cohen, 2018). Building on these insights, we hypothesized that participants would prefer honest feedback providers for themselves but would favour sensitive providers who adapt their feedback based on recipients' emotional resilience or prosocial liars when selecting providers for others, particularly for emotionally vulnerable individuals. The present research investigates not only how individuals evaluate the morality of different feedback providers but also how these moral judgements shape social preferences.

## OVERVIEW OF THE EXPERIMENTS

In this work, we examine how individuals judge and choose feedback providers. In Study 1, we investigated how people evaluate the morality, predictability, and trustworthiness of inconsistent feedback providers and compared them with two types of consistent providers. In the first control condition, we described someone who was consistently overly positive, whereas the second control condition described an individual who behaved consistently honestly. In the first experimental condition, we presented individuals who were inconsistent and misadjusted their feedback (inadequate feedback providers), and in the second experimental condition, we introduced providers who adjusted their feedback based on the recipient's emotional resilience (sensitive feedback providers). Study 2 compared preferences for the four types of feedback providers when participants were choosing for themselves versus for others, including individuals known to be vulnerable and less able to handle criticism.

We pre‐registered all studies: Study 1 https://aspredicted.org/92G_FNQ, Study 2 https://aspredicted.org/n6pc‐6fcg.pdf, Data, code and materials are available at https://osf.io/npg6k.

## STUDY 1

### Method

In Study 1, we examined whether individuals differ in their perceptions of feedback providers who offer honest versus overly positive feedback for poorly executed work.

#### Participants

Participants were located in the USA and were recruited via Prolific online research platform that received £9,15/hr. for participation in this study (median participation time was 7 min 52 s). We expected a small effect size of *η*_p_^2^ = 0.03 similar to reported by Walker et al. (2021). With *α* = .05, and 95% power, we aimed at reaching at least 286 participants in the analysed sample. Given that we expected that around 10% of participants may fail to respond correctly to the attention and comprehension check questions, we aimed to collect data from 315 participants. We managed to gather data from 327 participants. Data from 10 participants were excluded as they did not fill in the comprehension check and attention check questions. Further 21 participants were excluded as they incorrectly answered both comprehension check questions. The final sample consisted of 296 participants (145 women, 146 men, 2 – other, 2 – rather not say, 1 missing data). Ages ranged from 18 to 77 (*M*_*age*_ = 41.69, SD_age_ = 12.86). Sensitivity analysis using G*Power (Faul et al., 2007) suggested that with 95% power, *N* = 296, and four groups, we could detect small effects of *f* = 0.11 with *α* = .05.

#### Procedure and materials

Participants read a vignette about four individuals (e.g., Mary, Linda, Jen, and Susan) providing feedback on two poorly prepared dishes. The dishes were made separately by two people: Kate, who was described as handling negative feedback well, and Amy, who was described as handling negative feedback poorly. Gender of all characters in the vignettes was either female or male.

Each feedback provider (Mary, Linda, Jen, Susan) gave two private messages: one to Kate and one to Amy. The content of the feedback varied across four conditions:

- Prosocial Lies Control Condition: The character lied to both targets (e.g., ‘The dish you prepared is good’).
- Honesty Control Condition: The character told the truth to both targets (e.g., ‘The dish you prepared is not good’).
- Inadequate Feedback Provider Experimental Condition: The character lied to the target who handles feedback well and told the truth to the one who does not.
- Sensitive Feedback Provider Experimental Condition: The character lied to the target who handles feedback poorly and told the truth to the one who handles it well.

Each participant saw all four feedback providers (within‐subjects), presented in random order. After reading the messages, participants rated each feedback provider's moral traits (e.g., ‘Please rate what you think Linda is like’) using a 7‐point scale across four items (*α* = .80–.91; e.g., Bad–Good, Merciless–Empathetic, Immoral–Moral, Violent–Peaceful).

Participants also rated each feedback provider's predictability and trustworthiness (1 = *definitely disagree*, to 7 = *definitely agree*), and separately evaluated the appropriateness and impact of each feedback message (caused no harm–caused harm; brought benefits–brought no benefits; was a lie–was the truth; is expected in this situation–is unexpected) using a 7‐point scale with labels surrounding the endpoints of the scale. Finally, comprehension and attention check questions were presented. Complete materials presented to the participants can be accessed at the https://osf.io/npg6k.

### Results and discussion

#### Pre‐registered analysis

**FIGURE 1:**
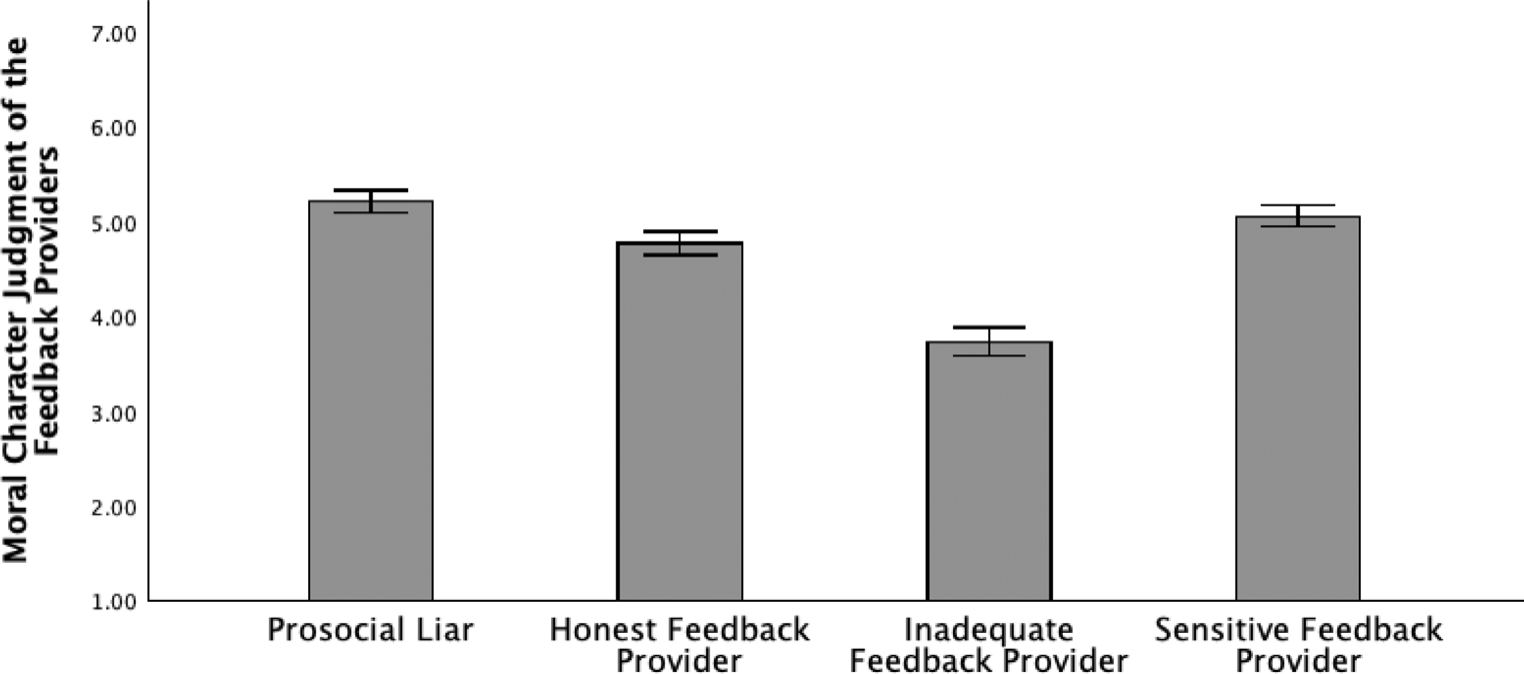
Comparison of Moral Character Judgement Towards Four Types of Feedback Providers: Prosocial Liar, Honest Feedback Provider, Inadequate Feedback Provider, and Sensitive Feedback Provider. Error bars represent 95% confidence intervals.

Our main goal was to test moral character judgement of individuals depending on whether they tell the truth or are prosocially dishonest to targets that differ in emotional resilience to negative feedback. We conducted a repeated measures ANOVA to test the main hypothesis with index of the four items measuring moral judgement as DV. Mean comparison throughout the article was performed with Bonferroni correction. The ANOVA results showed significant effect of type of feedback on moral judgement, *F*(2.64, 778.51) = 122.12, *p* < .001, *η*_p_^2^ = 0.29. However, the results differed from our hypotheses. Namely, post‐hoc mean comparison analyses showed that participants rated giving overly positive feedback to both targets as the most moral (*M* = 5.21, SD = 1.07). This rating did not differ from giving a lie to the non‐resilient target and the truth to the resilient one (*M* = 5.06, SD = 1.01), with a Bonferroni‐corrected *p* = .097. These ratings were followed by moral evaluations of giving honest feedback to both targets (*M* = 4.77, SD = 1.06), which differed significantly from the evaluation of the prosocial liar and the inadequate feedback provider at *p* < .001, as well as from the sensitive feedback provider (*p* = .002). The lowest moral evaluation was observed for the inadequate feedback provider (*M* = 3.73, SD = 1.32), which differed significantly from the evaluation of the prosocial liar at *p* < .001 (Figure 1).

In the Supplementary Materials we report moral character judgements of the feedback providers analysed in Study 2 and Study S1. The results consistently showed that the prosocial feedback provider was perceived as the most moral.

Although not pre‐registered, we also conducted repeated measures ANOVA with answers to just one item assessing how ‘good’ the behaviour was as DV. The results showed significant differences in the evaluation *F*(2.67, 786.47) = 75.28, *p* < .001, *η*_p_^2^ = 0.20. Comparison of means indicated that perception of the prosocial liar (*M* = 4.91, SD = 1.44) did not differ significantly from the evaluation of the honest feedback provider (*M* = 5.00, SD = 1.29), *p* = .999 and the sensitive feedback provider (*M* = 4.83, SD = 1.23), *p* = .999. The evaluation of the honest and the sensitive feedback providers did not differ significantly from one another, *p* = .511. The inadequate feedback provider (*M* = 3.65, SD = 1.52) was evaluated significantly worse from each of the three remaining feedback providers at *p* < .001.

#### Additional analysis

We examined whether gender of the participants and the evaluated characters affected the results. The gender of the feedback providers, *F*(1, 287) = 0.37, *p* = .544, *η*_p_^2^ < 0.01, of the participants, *F*(1, 287) = 1.19, *p* = .277, *η*_p_^2^ < 0.01, and their interaction, *F*(1, 287) = 2.70, *p* = .101, *η*_p_^2^ = 0.01 did not have an effect on the moral character judgement. Similarly, gender of the feedback providers *F*(2.64, 758.80) = 0.77, *p* = .497, *η*_p_^2^ = 0.01 and gender of the participants, *F*(2.64, 758.80) = 1.78, *p* = .156, *η*_p_^2^ = 0.01 did not interact with the experimental manipulation. Finally, the interaction of the gender of the feedback providers, the participants, and the experimental manipulation was also non‐significant, *F*(2.64, 758.80) = 0.95, *p* = .406, *η*_p_^2^ < 0.01. Detailed descriptive statistics are provided in Table S1 in the Supplementary Materials. We next tested how individuals rated predictability and trustworthiness of the characters giving feedback. We also examined evaluation of the feedback given to the two targets.

##### Predictability

We conducted a repeated measures ANOVA with predictability judgements as DV and type of feedback as IV. The results showed significant effect of type of feedback on predictability judgement, *F*(2.83, 834.57) = 86.45, *p* < .001, *η*_p_^2^ = 0.23. Participants rated as the most predictable the individual that gave honest feedback to both targets, that differed significantly from giving overly positive feedback to both targets, *p* = .002 and from giving a lie to non‐resilient target and the truth to the resilient one, *p* < .001. The latter two did not differ significantly from one another, *p* = .999. The least predictable was the individual that told a prosocial lie to the target that knew how to handle negative feedback and the truth to the target that had trouble with dealing with negative feedback. Evaluation of this character different significantly from the remaining ones at *p* < .001 (Table 1).

**TABLE 1:** Means and standard deviations for evaluation of predictability, trustworthiness, and perceived harm of feedback across different types of feedback providers.

Type of feedback provider	Predictability		Trustworthiness		Feedback harmfulness	
	*M*	SD	*M*	SD	*M*	SD
Prosocial liar	4.59	1.34	3.70	1.45	3.72	1.74
Honest feedback provider	4.94	1.19	5.53	1.21	3.62	1.25
Inadequate feedback provider	3.41	1.51	3.38	1.67	4.47	1.28
Sensitive feedback provider	4.48	1.22	4.19	1.35	3.16	1.28

##### Trustworthiness

We conducted a repeated measures ANOVA with trustworthiness judgements as DV and type of feedback as IV. The results showed significant effect of type of feedback on trust judgement, *F*(2.49, 735.14) = 157.99, *p* < .001, *η*_p_^2^ = 0.35. Similarly as with predictability judgements, participants rated as the most trustworthy the individual that gave honest feedback to both targets, which differed significantly from giving overly positive feedback to the non‐resilient to negative feedback target, while giving honest feedback to the resilient one, *p* < .001. Honest feedback provider was also evaluated as more trustworthy than the overly positive feedback provider, *p* < .001 and the inadequate feedback provider, *p* < .001. Trustworthiness towards the latter differed significantly from the prosocial liar, *p* = .006 and the sensitive feedback provider, *p* < .001The means and standard deviations are provided in Table 1.

##### Evaluation of the feedback

To analyse perceived harmfulness of the feedback given to the participants we first calculated the mean of the four evaluations across the two targets. We then conducted a repeated measures ANOVA with perceived harm caused by the feedback as the DV. The results indicated significant differences in perceived harm caused by the feedback depending on the type of feedback, *F*(2.41, 712.09) = 55.10, *p* < .001, *η*_p_^2^ = 0.16. Participants considered the most harmful the inadequate feedback (i.e., the truth to the non‐resilient target and a prosocial lie to the resilient one), which differed significantly from the perceived harm of the overly positive feedback to both of the targets, *p* < .001 and honest feedback to both of the targets, *p* < .001. The latter two means did not differ significantly, *p* = .999. Individuals evaluated as the least harmful the feedback provided by the sensitive individual (i.e., the truth to the resilient target and a prosocial lie to the non‐resilient one). This condition differed significantly from all the remaining ones at *p* < .001. The means and standard deviations can be found in Table 1.

##### Expectancy

Finally, we ran a repeated measures ANOVA with perception of expectancy of the given feedback as DV (with higher number indicating that it was perceived as unexpected). The results indicated significant differences in perceived expectancy of the feedback depending on its' type, *F*(2.74, 807.27) = 94.36, *p* < .001, *η*_p_^2^ = 0.24. Participants considered the most expected both the honest feedback passed to both of the targets, (*M* = 2.98, SD = 1.32) and the sensitive feedback (*M* = 3.20, SD = 1.25), *p* = .106. The condition of overly positive feedback (*M* = 4.27, SD = 1.45) did not differ significantly from the inadequate feedback condition (*M* = 4.49, SD = 1.39) in perceived expectancy, *p* = .331. All the other differences were significant at *p* < .001. In the Supplementary Materials we report results regarding benefits and truthfulness of feedback.

To examine a general pattern of correlations, we next calculated mean moral judgement of the four feedback providers, predictability, and trustworthiness assessment. We also calculated mean evaluation of harmfulness of the feedback. We conducted correlation analysis, which indicated that the strongest correlates of moral judgement were assessment of trustworthiness and feedback expectancy (Table 2).

**TABLE 2:** Correlation between evaluation of the characters (moral character judgement, predictability, trustworthiness) and the evaluation of harmfulness, truthfulness, how unexpected, and beneficial the feedback was.

Variable	1	2	3	4	5	6
1. Moral character judgement	—					
2. Predictable	.28***	—				
3. Trustworthy	.46***	.43***	—			
4. Feedback harmful	−.19***	−.10	−.27***	—		
5. Feedback beneficial	−.19***	−.21***	−.29***	.37***	—	
6. Feedback truthful	.19**	.13**	.43***	−.17**	−.23***	—
7. Feedback unexpected	−.29***	−.29***	−.26***	.30***	.20***	−.21***

** *p* < .01.

*** *p* < .001.

Finally, we also calculated an exploratory regression analysis, where we introduced moral character judgement as the outcome variable with the remaining variables (predictability, trustworthiness, harmfulness, benefits, truthfulness, and expectancy of feedback) as predictors. VIF values ranged from 1.22 to 1.58, indicating that multicollinearity was not a concern. The results indicated that when all the predictors were entered simultaneously into the analysis, only trustworthiness, *β* = .40, *t*(289) = 6.18, *p* < .001, 95% CI [0.21, 0.40] and unexpectancy, *β* = −.16, *t*(289) = −2.87, *p* = .004, 95% CI [−0.26, −0.05] judgements predicted significantly moral character judgements, *adjusted R* ^2^ = .24, *F*(6, 289) = 16.17, *p* < .001. Neither predictability *β* = .06, *t*(289) = 1.02, *p* = .309, 95% CI [−0.05, 0.15], harmfulness *β* = −.03, *t*(289) = −0.48, *p* = .630, 95% CI [−0.11, 0.07], benefits of the feedback *β* = −.03, *t*(289) = −0.55, *p* = .580, 95% CI [−0.12, 0.07] nor its' truthfulness *β* = −.04, *t*(289) = −0.64, *p* = .522, 95% CI [−0.13, 0.06] predicted moral judgement significantly.

The results of Study 1 reveal an intriguing pattern: prosocial liars are sometimes perceived as more moral than honest feedback providers. Even more remarkably, feedback providers who strategically adapt their honesty based on recipients' emotional resilience (‘sensitive’ providers) were judged as more moral than those who are consistently honest. These findings challenge the assumption that behavioural consistency is paramount in moral evaluations. Instead, they suggest that when making moral character judgements, people value a feedback provider's responsiveness to others' emotional needs over strict adherence to honesty. If people prioritized informational accuracy alone, which is maximized through honest feedback, we would expect the consistently honest feedback provider to receive the highest moral ratings. However, our results show that contextual sensitivity and consideration for others' psychological well‐being can outweigh the moral value of truthfulness in feedback scenarios.

## STUDY 2

### Method

We next wanted to examine how individuals select feedback providers based on the intended recipient. In a separate Study S1 presented in the Supplementary Materials, we asked participants to evaluate the same feedback providers as in Study 1 and then decide to choose one of the four feedback providers for themselves. The results indicated that the majority of participants (70%, *n* = 149), *χ* ^2^ (3, *N* = 213) = 231.83, *p* < .001, *ω* = 1.04 preferred an honest feedback provider.

In Study 2, we compared preferences when choosing a feedback provider for oneself versus for others, including those specifically identified as struggling with criticism. We hypothesized that individuals prefer honest feedback providers for themselves but favour sensitive providers (who adapt their feedback based on recipients' emotional resilience) or prosocial liars when selecting feedback providers for others, especially for emotionally vulnerable ones.

#### Participants

Participants were located in the USA Prolific online research platform users that received £6,51/hr. for participation in this study (median participation time was 6 min 55 s). Those that took part in Study 1 and the supplemental Study S1 could not participate in this study. We expected a small effect size *ω* = 0.20 similar to reported in Cantarero et al. (2022). With *α* = 0.05 and power of 0.95, we aimed at reaching at least 522 participants in the analysed sample. Assuming around 10% of participants may fail to respond correctly to the attention check questions, we aimed at collecting data from 574 participants. We managed to gather replies from 593 participants. Three replies from participants were excluded as they failed to reply to two the comprehension check and the attention check questions. The final sample consisted of 289 women, 280 men, 7 – undisclosed/other, 14 missing data. Ages ranged from 18 to 92 (*M*_*age*_ = 45.88, SD_*age*_ = 15.84). Sensitivity analysis using G*Power (Faul et al., 2009) suggested that with 95% power, *N* = 584, we could detect small effects of *ω* = 0.19 with *α* = .05.

#### Procedure and materials

The primary hypothesis was tested using a between‐subjects design with three conditions (self, unspecified other, vulnerable other), in which participants selected one of four feedback providers as the dependent variable.

Participants were presented with the same materials as in Study 1, yet without asking participants to evaluate the feedback with four questions (*caused no harm/caused harm; brought benefits/brought no benefits; was a lie/was the truth; is expected in this situation/is unexpected*). First participants evaluated how moral, good, peaceful, empathetic, predictable, and trustworthy each of the feedback providers were. Next, we asked participants to choose a feedback provider, which was our main DV. In the *Self* condition participants choose who they would like to get feedback from, in the *Unspecified other* condition, participants chose the feedback provider for another person and in the *Vulnerable other* condition who they would choose as the feedback provider for a person that struggles with feedback (‘Now imagine you/someone else/someone who takes negative feedback very personally and struggles to handle failure have prepared a dish that will be evaluated by either Mary, Linda, Jen or Susan’.). Participants were asked to choose one of four characters described previously: (1) Mary (who earlier told both Kate and Amy that their dishes were good); (2) Linda (who earlier told both Kate and Amy that their dishes were not good); (3) Jen (who earlier told Kate, who handles negative feedback well, that her dish was good and told Amy, who handles negative feedback badly, that her dish was not good); (4) Susan (who earlier told Kate, who handles negative feedback well, that her dish was not good and told Amy, who handles negative feedback badly, that her dish was good).

We added the same two comprehension check questions and an attention check question.

### Results and discussion

#### Pre‐registered analysis

**FIGURE 2:**
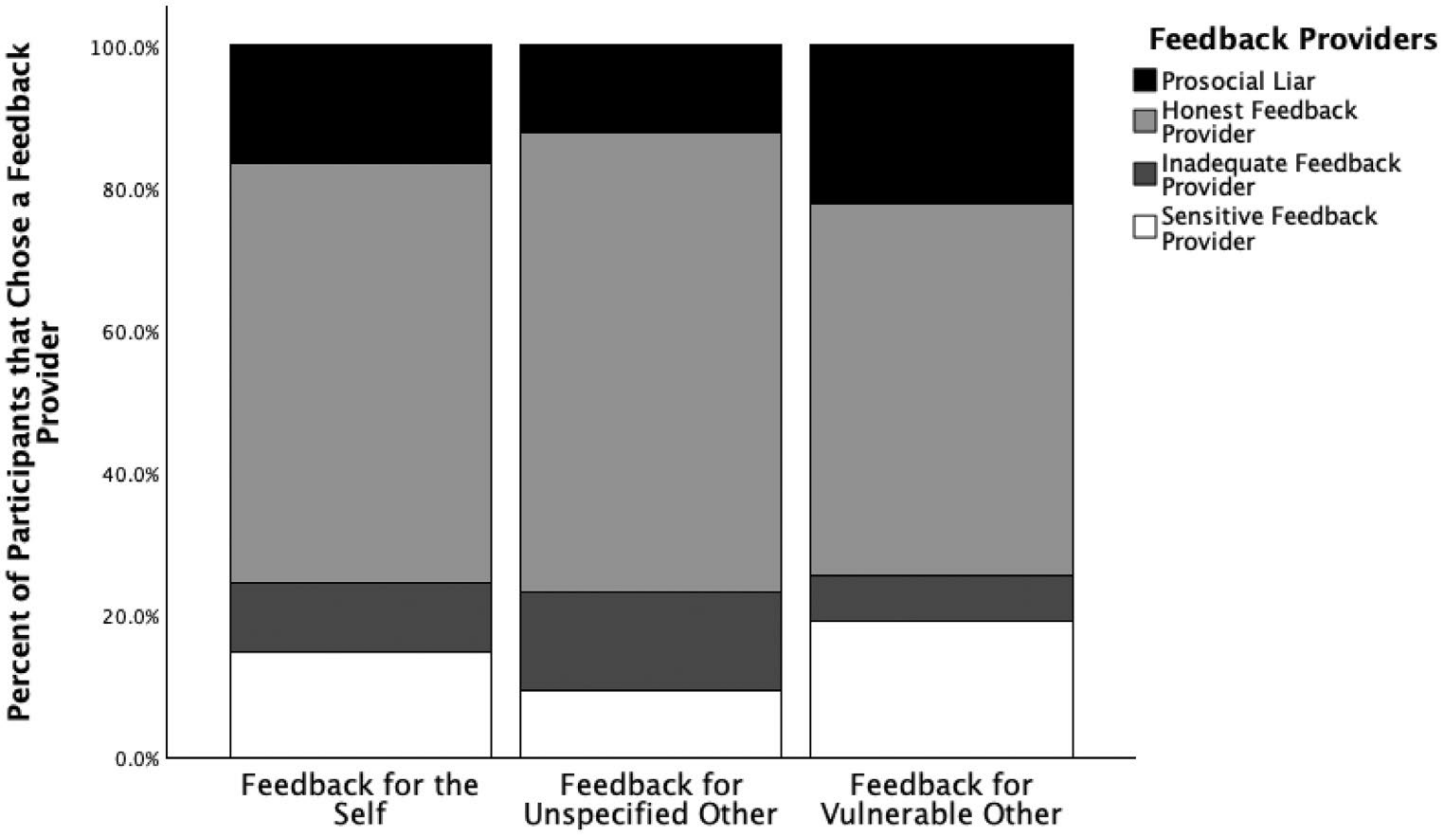
Comparison of preferences for four types of feedback providers (prosocial liar, honest feedback provider, inadequate feedback provider, and sensitive feedback provider) by feedback target (self, unspecified other, vulnerable other).

Our main goal was to test preference towards honest vs. dishonest feedback providers depending on whether the feedback is for the self, for another person or for another person that does not handle negative feedback well. We conducted *χ* ^2^ analysis and found a main effect of feedback receiver, *χ* ^2^ (6, *N* = 584) = 20.40, *p* = .002, *ω* = 0.18. Comparison of the distribution of proportions showed significant differences across the categories of type of feedback provider and to whom the feedback was intended. We did not find support for the hypothesis that individuals would differ in their preference towards feedback providers for the self versus for another person. Significant difference in proportion of choices arose between feedback for unspecified other person vs. feedback for another vulnerable person. Individuals chose a prosocial liar for the vulnerable other more frequently (22.3%, *n* = 43) than for unspecified other (12.4%, *n* = 24). Honest feedback provider was chosen least frequently for the vulnerable other (52.3%, *n* = 101), than for an unspecified other (64.4%, *n* = 125) or the self (58.9%, *n* = 116). The sensitive feedback provider was chosen most frequently for the vulnerable other (19.2%, *n* = 37), followed by unspecified other (9.3%, *n* = 18) and the self (14.7%, *n* = 29). We did not formulate any hypothesis regarding choosing the inadequate feedback provider. The results showed that this person was chosen most frequently for an unspecified other (13.9%, *n* = 27), followed with the self (9.6%, *n* = 19) and the vulnerable other (6.2%, *n* = 12). These results are displayed in Figure 2.

#### Additional analyses

To better interpret the results, we decided to conduct an exploratory multinomial logistic regression. We included the type of feedback recipient (self, other, or vulnerable other) as the IV and the feedback provider (prosocial liar, inadequate provider, sensitive provider, or honest provider) as the DV. The honest feedback provider was set as the reference category. The results indicated that the model was significant, *χ* ^2^ (6, *N* = 584) = 20.71, *p* = .002, indicating that feedback target significantly influenced choice of the feedback provider. However, the pseudo *R* ^2^ values were small (Cox & Snell *R* ^2^ = .035, Nagelkerke *R* ^2^ = .039, McFadden *R* ^2^ = .016). Given that the reference group was the honest feedback provider, the analysis examined the likelihood of selecting prosocial liar, inadequate provider, or sensitive provider relative to the honest feedback provider. We found that participants were significantly less likely to select a prosocial liar for an unspecified other compared with vulnerable other, *b* = −0.80, SE = 0.29, Wald(1) = 7.66, *p* = .006, OR = 0.45, 95% CI [0.26, 0.79]. This indicates that participants were 55% less likely to choose a prosocial liar for an unspecified other than for a vulnerable other. No significant difference were found in choosing a prosocial liar for the self, versus for a vulnerable other, *b* = −0.40, SE = 0.27, Wald(1) = 2.26, *p* = .133, OR = 0.67, 95% CI [0.40, 1.13].

We found no significant differences in choosing an inadequate feedback provider for the self vs. vulnerable other, *b* = 0.32, SE = 0.39, Wald(1) = 0.67, *p* = .414, OR = 1.38, 95% CI [0.64, 2.98] or for an unspecified other compared with vulnerable other, *b* = 0.60, SE = 0.37, Wald(1) = 2.58, *p* = .108, OR = 1.82, 95% CI [0.88, 3.77].

Participants were less likely to choose a sensitive feedback provider for an unspecified other compared with a vulnerable other, *b* = −0.93, SE = 0.32, Wald(1) = 8.68, *p* = .003, OR = 0.39, 95% CI [0.21, 0.73], suggesting that participants were 61% less likely to choose a sensitive feedback provider when the recipient was an unspecified other than when the recipient was a vulnerable other. We found no significant difference in choosing a sensitive feedback provider for the self, compared with the vulnerable other, *b* = −0.38, SE = 0.28, Wald(1) = 1.82, *p* = .177, OR = 0.68, 95% CI [0.39, 1.19].

The results of this study indicate that individuals generally prefer an honest feedback provider. Notably, participants were more likely to prefer a prosocial liar or a sensitive feedback provider when selecting a feedback provider for someone who struggles with criticism, compared with an unspecified other. This suggests that individuals are more willing to protect others from harsh truth only when the recipient's vulnerability is explicitly signalled.

## GENERAL DISCUSSION

The results of our studies indicate that a prosocial liar can sometimes be perceived as more moral than an honest feedback provider. Interestingly, an inconsistent yet sensitive feedback provider, the one who adapts their honesty based on recipients' emotional resilience, was not penalized for their inconsistency; rather, Study 1 revealed that such individuals were perceived as even more moral than a consistently honest feedback provider. However, Study 2 revealed that honest feedback providers were preferred over sensitive feedback providers when individuals selected feedback for themselves. Additionally, we found that individuals exert a higher preference to choose a sensitive feedback provider or a prosocial liar for another person that handles criticism badly compared with when someone does not signal vulnerability.

Although previous research suggests that individuals underestimate others' desire for feedback (Abi‐Esber et al., 2022), our findings indicate that an honest feedback provider is preferred when individuals are not responsible for delivering the feedback themselves. Our findings support the notion that truth is often the default and most desired option, yet prosocial liars can still benefit from positive moral character judgements. Prior research suggests that predictability and consistency are key determinants of moral character perception (Turpin et al., 2021; Walker et al., 2021). However, our results suggest that individuals may tolerate ‘inconsistent predictability’ when behaviour is appropriately responsive to others' needs. This suggests that moral character judgements do not always align with perceptions of predictability or trustworthiness. Instead, individuals may prioritize contextual sensitivity over rigid consistency, when evaluating moral character.

Our findings align with previous research showing that liars can sometimes be perceived as more moral than truth‐tellers (Levine & Schweitzer, 2014). However, consistent with self‐verification theory (Kwang & Swann, 2010), on average two out of three individuals still prefer accurate feedback providers over those who offer overly positive feedback. Hence, some people see prosocial liars as more moral but still prefer to receive honest feedback. This mismatch between moral character judgements and preferences for feedback providers is intriguing and warrants further investigation. Additionally, our findings contribute to the literature on the proportionality moral foundation, which posits that moral judgements do not always favour equal treatment but instead adjust according to individuals' specific merits and circumstances (Atari et al., 2023). Our results further suggest that moral character judgements are shaped by how well individuals respond to the specific needs of others. In this context, dishonesty that is sensitive to others' needs may be socially approved and perceived as morally justified, even though it involves deception.

Exploratory analyses conducted in Study 1 suggested that both trustworthiness and the extent to which the behaviour of the feedback provider was expected emerged as the strongest predictors of moral character judgement. Our findings align with research by Levine and Schweitzer (2015), who showed that prosocial lying can enhance trust by signalling benevolent intentions, an important factor in the formation of social bonds. Moreover, as Levine (2022) argues, social norms do not always prioritize honesty above all else; in some situations, individuals may even be expected to violate the norm of honesty, particularly when telling the truth could cause unnecessary harm, such as when the recipient is sensitive to negative feedback. The pattern of means indicated that both consistently providing honest feedback and tailoring feedback: being honest with a resilient target while telling a prosocial lie to a vulnerable one, were perceived as the most expected responses. This suggests that people anticipate others to uphold both the norm of honesty and the norm of benevolence in their moral evaluations.

### Limitations and directions of future studies

One limitation of this research is that we assessed hypothetical preferences rather than actual behaviour. Future research should investigate whether individuals' stated preferences align with their real‐world choices of feedback providers. Misalignment between stated preferences and actual behaviour is a known issue in the field, with large changes to intentions translate into much weaker changes in behaviour (Webb & Sheeran, 2006). Considering these limitations, our results should be interpreted cautiously. Additionally, the study relied on a single scenario, which could have been perceived by some participants as having limited social relevance. While the use of a familiar, everyday dilemma was intended to enhance ecological validity, we acknowledge that focusing on a single scenario constrains the generalizability of the findings.

Several contextual factors not explored in this study may influence feedback preferences. For example, previous research suggests that rejection threat can shift preferences towards accurate feedback (Kwang & Swann, 2010), which could be an important avenue for future research. In this study, we did not collect detailed demographic information about participants (e.g., occupational role or rank). It is possible that such characteristics could moderate how feedback providers are evaluated, which would be a valuable direction for future research.

Our target evaluations did not adopt the classic model of character judgement, which distinguishes between agency and communion (Abele & Wojciszke, 2014). Prior research has shown that communion is the predominant dimension in judging others (Wojciszke et al., 1998), and the classical conceptualization of communion is often further divided into morality and sociability (Brambilla et al., 2021). Morality, in this context, reflects the extent to which social targets behave appropriately, and are perceived as honest and trustworthy (e.g., Brambilla & Leach, 2014). Given that violating the norm of honesty should primarily impact the moral domain, we focused specifically on moral character judgements. However, we acknowledge that examining the remaining evaluative dimensions in future research could provide a more nuanced understanding of the phenomenon.

In this study, we did not examine the underlying psychological processes driving the observed effects. Future research should investigate whether reduced empathy, heightened rigidity, or (un)intentional misalignment with social norms may underlie less favourable moral character judgements of the honest feedback provider. Additionally, our study suggests that the way individuals adhere to the equality and proportionality moral foundations makes a meaningful difference. In the eyes of social perceivers, treating others equally honestly can diverge from treating them equally dishonestly, even when dishonesty serves the target's benefit. Acting in accordance with the proportionality foundation may be perceived as similarly appropriate as adhering to the equality foundation, provided it aligns with contextual demands. Future studies should further examine the tension between these two moral foundations and consider applying the MFQ‐2 (Atari et al., 2023; Zakharin & Bates, 2023) to capture individual differences in moral foundations and better illuminate the nuanced mechanisms underlying these effects.

Moreover, perceptions of feedback accuracy play a crucial role in feedback selection. Research indicates that individuals tend to prefer feedback that is accurate rather than simply consistent with their self‐views (Szumowska et al., 2022). Future studies could examine whether moral character judgements and feedback preferences depend on subjective perceptions of performance quality and the importance of the task (both for the self and for others).

Temporal perspective may play a pivotal role in how feedback providers are judged. According to Construal Level Theory (CLT; Trope & Liberman, 2010), greater temporal distance from an event typically promotes more abstract, high‐level thinking, whereas shorter temporal distance fosters concrete, detail‐oriented processing. Abstract construals emphasize overarching goals and central features, while concrete construals focus on specific, contextual details. As a result, temporal distance systematically influences how individuals interpret and evaluate events, which can, in turn, affect subsequent judgements and decisions (Agerström et al., 2012; Alper, 2020). In the long run, honest feedback may be valued more highly as it contributes to performance improvement and achieving central goals. Thus, individuals might show a greater preference for honest feedback providers when long‐term outcomes are salient. Conversely, in the early stages of an activity, positive, perhaps even exaggerated, feedback might serve as a motivational tool, making those who provide it more favourably judged in such contexts. Future research should investigate how feedback preferences and moral character judgements shift based on temporal considerations.

## CONCLUSION

Taken together, our studies suggest that individuals are highly sensitive to social cues when evaluating (dis)honesty in feedback provision. Rather than being penalized, inconsistent behaviour is accepted and even rewarded when it aligns with social expectations and contextual needs. Only when inconsistency contradicts social reality is it perceived as less moral. These findings contribute to a nuanced understanding of how individuals judge and select feedback providers, highlighting the complex interplay between honesty, prosociality, and contextual sensitivity.

## AUTHOR CONTRIBUTIONS

**Katarzyna Cantarero:** Conceptualization; investigation; funding acquisition; writing – original draft; methodology; visualization; writing – review and editing; formal analysis; project administration; data curation; supervision; resources. **Michał Białek:** Conceptualization; supervision; writing – original draft; writing – review and editing; methodology.

## CONFLICT OF INTEREST STATEMENT

The authors have no conflict of interest to declare.

## ETHICS STATEMENT

Across all studies, we report all manipulations, measures, and exclusions. The Faculty Committee of Ethics of Scientific Research at SWPS University (approval number 01/P/12/2020) approved all studies. Participants gave their informed consent prior to taking part in the studies. We pre‐registered the two studies: Study 1 https://aspredicted.org/92G_FNQ, Study 2 https://aspredicted.org/n6pc‐6fcg.pdf, Data, code and materials are available at https://osf.io/npg6k.

## DECLARATION OF GENERATIVE AI AND AI‐ASSISTED TECHNOLOGIES IN THE WRITING PROCESS

During the preparation of this work the authors used ChatGPT‐4 to improve the readability and language of the manuscript. After using this tool, the authors reviewed and edited the content as needed and take full responsibility for the content of the published article.

## STATEMENT OF CONTRIBUTION

- Predictability shapes moral judgement, but strategic inconsistency can appear more moral.
- People value sensitivity to others' needs sometimes more than rigid honesty in moral judgement.
- Inconsistency is judged as immoral only when it contradicts social reality.

## Supporting information


Data S1.

## Data Availability

The data that support the findings of this study are openly available in osf.io at https://osf.io/npg6k.
