# Supplementary material for: Selective (dis)honesty: Choosing overly positive feedback only when the truth hurts
Source: Br J Soc Psychol. 2025 Nov 23;65(1):e70020. doi: 10.1111/bjso.70020 (PMC12641110; doi:10.1111/bjso.70020)
Supplement: Supplementary file 1 — Data S1. [file BJSO-65-0-s001.docx]

**Supplementary Materials**

**Selective (Dis)honesty: Choosing Overly Positive Feedback Only When the Truth Hurts**

**Study S1**

**Method**

In Study S1, we wanted to test if people use the information on the tendency to give honest vs overinflated positive feedback when deciding who to choose as their own feedback provider. On the one hand self-enhancement motivation relates with individuals’ desire to increase positivity of their self-views (e.g., Leary, 2007; Sedikides & Gregg, 2008), which could favor choosing a prosocial liar that could secure positive self-views. However, self-verification motivation posits that individuals prefer accurate information, consistent with self-views (e.g., Swann, 2012; Kwang & Swann, 2010), which suggests that an honest feedback provider should be preferred. We thought that individuals may actually favor the sensitive feedback provider (gives overly positive feedback to the target that handles criticism badly and honest feedback to the one that handles criticism well) due to potentially benefitting both from self-enhancement and self-verification motivation in that individuals could anticipate that they would be given honest feedback or a prosocial lie when needed. We expected this preference to be followed by choosing the truth-teller, next the prosocial liar and the insensitive feedback provider (gives overly positive feedback to the target that handles criticism well and honest feedback to the one that handles criticism badly). The study was preregistered at <https://aspredicted.org/JVX_XDY>,

***Participants***

Participants were located in the USA Prolific online research platform users that received £9,05/hr for participation in this study (median participation time was 5min 38 sec). Those that took part in the previous study could not participate in Study 2. We expected an effect size ω = 0.30. With alpha = 0.05, power of 0.95, we aimed at reaching at least 191 participants in the analyzed sample. Given that we expected that around 10% of participants may fail to respond correctly to the attention check questions, we aimed at collecting data from 210 participants. We managed to gather replies from 213 participants. No replies from participants were excluded from the data file as there was no person that failed two the comprehension check and the attention check questions. The final sample consisted of 148 women, 56 men, 6 – undisclosed/other, 3 missing data. Ages ranged from 19 to 84 (*M_age_* = 40.01, *SD_age_* = 12.34). Sensitivity analysis using G*Power (Faul et al., 2009) suggested that with 95% power, *N* = 213, we could detect small effects of ω = 0.28 with α = .05.

***Procedure*** ***and Materials***

Participants were presented with the same materials as in Study 1 with the exception that we did not ask to evaluate the feedback with four questions (*caused no harm/caused harm; brought benefits/brought no benefits; was a lie/was the truth; is expected in this situation/is unexpected*). Next, participants were asked to choose who they would like to get feedback from ("Now imagine you've prepared a dish and want feedback on whether it's good or bad. Who would you choose to give you that feedback?") and the four names of the characters described earlier were available to choose from: 1. Mary (who earlier told both Kate and Amy that their dishes were good); 2. Linda (who earlier told both Kate and Amy that their dishes were not good); 3. Jen (who earlier told Kate, who handles negative feedback well, that her dish was good and told Amy, who handles negative feedback badly, that her dish was not good); 4. Susan (who earlier told Kate, who handles negative feedback well, that her dish was not good and told Amy, who handles negative feedback badly, that her dish was good).

We added the same two comprehension check questions and an attention check question.

***Results and Discussion***

**Pre-registered Analysis.** Our main goal was to test preference towards honest vs dishonest feedback providers. We conducted chi2 analysis and found that the majority of individuals preferred the honest feedback provider (70%, *n* = 149), followed by the sensitive feedback provider (14%, *n* = 30), the prosocial liar (9%, *n* = 19) and the inadequate feedback provider was least preferred (7%, *n* = 15), *χ*^2^ (3, *N* = 213) = 231.83, *p* < .001,  *ω* = 1.04.

**Additional analysis.** We conducted analysis to test moral character judgment of individuals depending on whether they tell the truth or are prosocially dishonest to targets that differ in emotional resilience to negative feedback. We also tested how individuals rated predictability and trustworthiness of the characters giving feedback.

***Moral judgment.*** We conducted a repeated measures ANOVA to test the hypothesis with index of the four items measuring moral judgment as DV (α = .80-.89, *Bad/Good, Immoral/Moral, Violent/Peaceful, Merciless/Empathetic*). The ANOVA results showed significant effect of type of feedback on moral judgment, *F*(2.71, 575.38) = 64.38, *p* < .001, η_p_^2^ = .23. The results were similar to those in Study 1. Namely, participants rated as the most moral giving overly positive feedback to both targets (*M* = 5.17, *SD* = 1.06), that differed from giving a lie to non-resilient target and the truth to the resilient one (*M* = 4.96, *SD* = 1.02), *p* = .020. This evaluation did not differ significantly from the moral judgment of giving honest feedback to both targets (*M* = 4.85, *SD* = 1.00), *p* = .965. The least moral was the character in the Inadequate feedback condition (*M* = 4.03, *SD* = 1.28). The remaining differences were statistically significant at *p* ≤ .001.

***Predictability.*** We conducted a repeated measures ANOVA with predictability judgments as DV and type of feedback as IV.  The results showed significant effect of type of feedback on predictability judgment, *F*(2.69, 569.42) = 40.26, *p* < .001, η_p_^2^ = .16. Similar to Study 1, participants rated as the most predictable the individual that gave honest feedback to both targets (*M* = 4.84, *SD* = 1.14), that differed significantly from giving overly positive feedback to both targets (*M* = 4.54, *SD* = 1.24), *p* = .035 and from giving a lie to non-resilient target and the truth to the resilient one (*M* = 4.32, *SD* = 1.18), *p* < .001. The latter two did not differ significantly from one another, *p* = .111. The least predictable was the individual that told a prosocial lie to the target that knew how to handle negative feedback and the truth to the target that had trouble with dealing with negative feedback (*M* = 3.69, *SD* = 1.46). Evaluation of this character different significantly from the remaining ones at *p* < .001.

***Trustworthiness****.* We conducted a repeated measures ANOVA with trustworthiness judgments as DV and type of feedback as IV.  The results showed significant effect of type of feedback on trust judgment, *F*(2.55, 540.54) = 102.44, *p* < .001, η_p_^2^ = .33. Similarly as with predictability judgments and as in Study 1, participants rated as the most trustworthy the individual that gave honest feedback to both targets (*M* = 5.39, *SD* = 1.15), followed by giving overly positive feedback to the non-resilient to negative feedback target, while giving honest feedback to the resilient one (*M* = 4.00, *SD* = 1.37). Third trustworthiness judgment was passed to the person that told overly positive feedback to both of the targets (*M* = 3.61, *SD* = 1.40). The least trustworthy was the inadequate feedback provider (*M* = 3.46, *SD* = 1.59), whose trustworthiness judgments did not differ from judging the prosocial liar significantly, *p* > .999. The remaining differences between the means were significant at *p* < .001.

To examine a general pattern of correlations, we next calculated mean moral judgment of the four characters, predictability and trustworthiness assessment. We conducted correlation analysis, which indicated that moral judgment of the characters were related positively to trustworthiness, *r*(213) = .52, *p* < .001 and predictability *r*(213) = .15, *p* = .024. The latter two were also positively related, *r*(213) = .36, *p* < .001.

This suggests that although Study 1 indicated that individuals perceive the prosocial liar as more moral than the honest one and the feedback given by the sensitive feedback provider as the least harmful, individuals nevertheless prefer an honest feedback provider for themselves.

**Additional Analysis Study 1 and Study 2**

**Study 1**

We conducted a repeated measures ANOVA with perceived benefits (which was coded with higher number indicating no benefits) of the feedback as the DV. The results indicated significant differences in perceived benefits related to the type of feedback, *F*(2.19, 644.93) = 82.10, *p* < .001, η_p_^2^ = .22. Participants considered the least beneficial the overly positive feedback passed to both of the targets, (*M* = 4.87, *SD* = 1.75), which did not differ significantly from the condition with inadequate feedback (*M* = 4.77, *SD* = 1.33), *p* > .999, but differed significantly from the condition of sensitive feedback (*M* = 3.71, *SD* = 1.20), *p* < .001 and all honesty (*M* = 3.40, *SD* = 1.55), *p* < .001. The latter two differed at *p* = .013. These results showed that all honesty followed with sensitive feedback were perceived as the most beneficial.

We also conducted a repeated measures ANOVA with perceived truthfulness of the feedback as the DV. The results indicated significant differences in perceived truthfulness of the feedback depending on its’ type, *F*(2.35, 691.64) = 466.15, *p* < .001, η_p_^2^ = .61. Participants considered the most deceitful the overly positive feedback passed to both of the targets, (*M* = 2.19, *SD* = 1.69), which differed significantly from the condition with honest feedback (*M* = 6.24, *SD* = 1.26), *p* < .001, the latter was perceived as the most truthful one. The condition of sensitive feedback (*M* = 4.18, *SD* = 1.13) and inadequate feedback (*M* = 4.13, *SD* = 1.44) did not differ between each other in perceived truthfulness, *p* > .999. All the other differences were significant at *p* < .001.

We examined whether gender of the participants and the evaluated characters affected the results. The gender of the feedback providers, *F*(1, 287) = 0.37, *p* = .544, η_p_^2^ < .01, of the participants, *F*(1, 287) = 1.19, *p* = .277, η_p_^2^ < .01, and their interaction, *F*(1, 287) = 2.70, *p* = .101, η_p_^2^ = .01 did not have a main effect on the moral character judgment. Similarly, gender of the feedback providers *F*(2.64, 758.80) = 0.77, *p* = .497, η_p_^2^ = .01 and gender of the participants, *F*(2.64, 758.80) =  1.78, *p* = .156, η_p_^2^ = .01 did not interact with the experimental manipulation. Finally, the interaction of the gender of the feedback providers, the participants and the experimental manipulation was also non-significant, *F*(2.64, 758.80) =  0.95, *p* = .406, η_p_^2^ < .01.

**Table S1**

*Moral Character Judgment of the Feedback Provider Depending on Their Gender and on the Gender of the Participant*

| Fedback Provider | Character’s Gender | Gender of the Participant | *M* | *SD* |
| --- | --- | --- | --- | --- |
| Prosocial Liar | Female | Female | 5.53 | 0.98 |
|  |  | Male | 5.14 | 1.12 |
|  |  | Total | 5.37 | 1.05 |
|  | Male | Female | 5.06 | 1.11 |
|  |  | Male | 5.06 | 1.03 |
|  |  | Total | 5.06 | 1.06 |
|  | Total | Female | 5.31 | 1.06 |
|  |  | Male | 5.10 | 1.07 |
|  |  | Total | 5.21 | 1.07 |
| Honest Fedback Provider | Female | Female | 4.83 | 1.00 |
|  |  | Male | 4.85 | 1.13 |
|  |  | Total | 4.84 | 1.05 |
|  | Male | Female | 4.74 | 1.02 |
|  |  | Male | 4.67 | 1.14 |
|  |  | Total | 4.71 | 1.08 |
|  | Total | Female | 4.79 | 1.01 |
|  |  | Male | 4.75 | 1.13 |
|  |  | Total | 4.77 | 1.07 |
| Inadequate Feedback Provider | Female | Female | 3.74 | 1.32 |
|  |  | Male | 3.64 | 1.35 |
|  |  | Total | 3.69 | 1.33 |
|  | Male | Female | 3.69 | 1.30 |
|  |  | Male | 3.92 | 1.30 |
|  |  | Total | 3.81 | 1.30 |
|  | Total | Female | 3.71 | 1.31 |
|  |  | Male | 3.79 | 1.32 |
|  |  | Total | 3.75 | 1.32 |
| Sensitive Feedback Provider | Female | Female | 5.19 | 0.96 |
|  |  | Male | 4.94 | 1.12 |
|  |  | Total | 5.09 | 1.04 |
|  | Male | Female | 4.93 | 1.12 |
|  |  | Male | 5.10 | 0.87 |
|  |  | Total | 5.02 | 1.00 |
|  | Total | Female | 5.08 | 1.04 |
|  |  | Male | 5.03 | 0.99 |
|  |  | Total | 5.05 | 1.02 |

**Study 2**

We conducted analysis to test moral character judgment of individuals depending on whether they tell the truth or are prosocially dishonest to targets that differ in emotional resilience to negative feedback. We also tested how individuals rated predictability and trustworthiness of the characters giving feedback.

***Moral judgment.*** We conducted a repeated measures ANOVA to test the main hypothesis with index of the four items measuring moral judgment as DV (α = .80-.89, *Bad/Good, Immoral/Moral, Violent/Peaceful, Merciless/Empathetic*). The ANOVA results showed significant effect of type of feedback on moral judgment, *F*(2.73, 1595.09) = 137.23, *p* < .001, η_p_^2^ = .19. The results were similar to those in Study 1. Namely, participants rated as the most moral giving overly positive feedback to both targets (*M* = 5.26, *SD* = 1.09), that differed from giving a lie to non-resilient target and the truth to the resilient one (*M* = 4.90, *SD* = 1.10), *p* < .001. This time the evaluation of the prosocial liar as feedback provider differed significantly from the moral judgment of giving honest feedback to both targets (*M* = 4.81, *SD* = 1.09), *p* < .001. The evaluation of the honest feedback provider and the sensitive feedback provider did not differ significantly from one another, *p* = .268. The least moral was the inadequate feedback provider (*M* = 4.14, *SD* = 1.34). The remaining differences were statistically significant at *p* < .001.

***Predictability.*** We conducted a repeated measures ANOVA with predictability judgments as DV and type of feedback as IV.  The results showed significant effect of type of feedback on predictability judgment, *F*(2.87, 1677.20) = 78.61, *p* < .001, η_p_^2^ = .12. Similar to Study 1and Study S1, participants rated as the most predictable the individual that gave honest feedback to both targets (*M* = 4.79, *SD* = 1.25), that differed significantly from giving overly positive feedback to both targets (*M* = 4.48, *SD* = 1.33), *p* < .001 and from giving a lie to non-resilient target and the truth to the resilient one (*M* = 4.30, *SD* = 1.32), *p* < .001. The latter two differ significantly from one another, *p* = .041. The least predictable was the individual that told a prosocial lie to the target that knew how to handle negative feedback and the truth to the target that had trouble with dealing with negative feedback (*M* = 3.75, *SD* = 1.46). Evaluation of this character different significantly from the remaining ones at *p* < .001.

***Trustworthiness****.* We conducted a repeated measures ANOVA with trustworthiness judgments as DV and type of feedback as IV.  The results showed significant effect of type of feedback on trust judgment, *F*(2.64, 1544.10) = 200.95, *p* < .001, η_p_^2^ = .26. Similarly as with predictability judgments and as in Study 1 and Study S1, participants rated as the most trustworthy the individual that gave honest feedback to both targets (*M* = 5.32, *SD* = 1.14), followed by giving overly positive feedback to the non-resilient to negative feedback target, while giving honest feedback to the resilient one (*M* = 4.11, *SD* = 1.44). The latter judgment differed significantly from trustworthiness judgment was passed to the person that told overly positive feedback to both of the targets (*M* = 3.89, *SD* = 1.47), *p* = .005. The least trustworthy was the inadequate feedback provider (*M* = 3.62, *SD* = 1.62), whose trustworthiness judgments differed from judging the prosocial liar significantly. The remaining differences between the means were significant at *p* < .001.

To examine a general pattern of correlations, we next calculated mean moral judgment of the four characters, predictability and trustworthiness assessment. We conducted correlation analysis, which indicated that moral judgment of the characters were related positively to trustworthiness, *r*(590) = .46, *p* < .001 and predictability *r*(590) = .15, *p* < .001. The latter two were also positively related, *r*(590) = .28, *p* < .001.

**References**

Leary, M. R. (2007). Motivational and emotional aspects of the self. *Annual Review of Psychology*, *58*(1), 317-344. <https://doi.org/10.1146/annurev.psych.58.110405.085658>

Sedikides, C., & Gregg, A. P. (2008). Self-Enhancement: Food for Thought. *Perspectives on Psychological Science*, *3*(2), 102–116. <https://doi.org/10.1111/j.1745-6916.2008.00068.x>

Swann, W. B., Jr. (2012). Self-verification theory. In P. A. M. Van Lange, A. W. Kruglanski, & E. T. Higgins (Eds.), *Handbook of theories of social psychology* (pp. 23–42). Sage Publications Ltd. [https://doi.org/10.4135/9781446249222.n27](https://psycnet.apa.org/doi/10.4135/9781446249222.n27)
